# Low Dose Organochlorine Pesticides and Polychlorinated Biphenyls Predict Obesity, Dyslipidemia, and Insulin Resistance among People Free of Diabetes

**DOI:** 10.1371/journal.pone.0015977

**Published:** 2011-01-26

**Authors:** Duk-Hee Lee, Michael W. Steffes, Andreas Sjödin, Richard S. Jones, Larry L. Needham, David R. Jacobs

**Affiliations:** 1 Department of Preventative Medicine, School of Medicine, Kyungpook National University, Daegu, Korea; 2 Department of Laboratory Medicine and Pathology, School of Medicine, University of Minnesota, Minneapolis, Minnesota; 3 Organic Analytical Toxicology Branch (OAT), Division of Laboratory Sciences (DLS), National Center for Environmental Health (NCEH), Centers for Disease Control and Prevention (CDC), Atlanta, Georgia, United States of America; 4 Division of Epidemiology, School of Public Health, University of Minnesota, Minneapolis, Minnesota; 5 Department of Nutrition, University of Oslo, Oslo, Norway; East Carolina University, United States

## Abstract

**Background:**

There is emerging evidence that background exposure to persistent organic pollutants (POPs) are important in the development of conditions predisposing to diabetes as well as of type 2 diabetes itself. We recently reported that low dose POPs predicted incident type 2 diabetes in a nested case-control study. The current study examined if low dose POPs predicted future adiposity, dyslipidemia, and insulin resistance among controls without diabetes in that study.

**Methodology/Principal Findings:**

The 90 controls were diabetes-free during 20 years follow-up. They were a stratified random sample, enriched with overweight and obese persons. POPs measured in 1987-88 (year 2) sera included 8 organochlorine (OC) pesticides, 22 polychlorinated biphenyls (PCBs), and 1 polybrominated biphenyl (PBB). Body mass index (BMI), triglycerides, HDL-cholesterol, LDL-cholesterol, and homeostasis model assessment value for insulin resistance (HOMA–IR) were study outcomes at 2005-06 (year 20). The evolution of study outcomes during 18 years by categories of serum concentrations of POPs at year 2 was evaluated by adjusting for the baseline values of outcomes plus potential confounders. Parallel to prediction of type 2 diabetes, many statistically significant associations of POPs with dysmetabolic conditions appeared at low dose, forming inverted U-shaped dose-response relations. Among OC pesticides, p,p'-DDE most consistently predicted higher BMI, triglycerides, and HOMA-IR and lower HDL-cholesterol at year 20 after adjusting for baseline values. Oxychlordane, trans-nonachlor, and hexachlorobenzene also significantly predicted higher triglycerides. Persistent PCBs with ≥7 chlorides predicted higher BMI, triglycerides, and HOMA-IR and lower HDL-cholesterol at year 20 with similar dose-response curves.

**Conclusions/Significance:**

Simultaneous exposure to various POPs in the general population may contribute to development of obesity, dyslipidemia, and insulin resistance, common precursors of type 2 diabetes and cardiovascular diseases. Although obesity is a primary cause of these metabolic abnormalities, POPs exposure may contribute to excess adiposity and other features of dysmetabolism.

## Introduction

There is emerging evidence that background exposure to persistent organic pollutants (POPs) may play an important role in the development of type 2 diabetes. Recently, we reported that serum concentrations of some POPs, in particular organochlorine (OC) pesticides and polychlorinated biphenyls (PCBs), predicted incident type 2 diabetes 1in a nested case-control study[1], concordant with cross-sectional findings in the U.S. general population[2], [3]. Among OC pesticides, Chlordane compounds were most strongly associated with type 2 diabetes while among PCBs, persistent congeners mainly predicted type 2 diabetes. Importantly, POPs at low doses, similar to current population exposure levels, increased the risk of diabetes while higher dose POPs did not increase the risk[1], similar to known low dose effects of endocrine disruptors[4], [5]. Incident type 2 diabetes was also associated with p,p'-DDE, but not PCBs, in 471 Great Lakes Sport Fish Consumers[6].

In the current paradigm for type 2 diabetes, obesity results from energy imbalance, insulin resistance from obesity, and exhaustion of pancreatic beta cells from overproduction of insulin to compensate for insulin resistance, which ultimately progresses to diabetes[7]. In fact, background exposure to OC pesticides and PCBs was cross-sectionally associated with insulin resistance and dyslipidemia even among people without diabetes[8], [9]. Also, p,p'-DDE exposure in utero was linked to obesity in adult female offspring of the Michigan fisheater cohort[10]. Together, these findings suggest that low dose OC pesticides and PCBs may play a role in dysmetabolism and diabetogenesis even in its developmental stages.

One recent animal study strongly supported this concept. Rats exposed to POPs through the consumption of fish oil contaminated with various POPs developed abdominal obesity, hepatosteatosis, and insulin resistance[11]. Also, POPs down-regulated insulin-induced gene-1 and Lpin1, two master regulators of lipid homeostasis[11]. We know of no previous prospective study of whether human exposure to POPs predicts obesity, insulin resistance, and associated metabolic disorders. Therefore, we examined the relationship of serum concentrations of POPs with adiposity, dyslipidemia, and insulin resistance among people confirmed to be diabetes-free on 5 occasions over 20 years, using the control group of our nested case-control study[1]. Based on previous findings, we hypothesized stronger dysmetabolic effects at low dose POPs.

## Methods

### Source population

CARDIA focuses on cardiovascular disease risk development in participants initially aged 18 to 30 years. In brief, 5,115 African American and white participants were recruited at baseline in 1985–1986 (year 0)[12] and follow-up examinations were completed at years 2, 5, 7, 10, 15, and 20 (2005-06) in 91%, 86%, 81%, 79%, 74%, and 72% respectively, of survivors.

The study was approved by the University of Minnesota Institutional Review Board, as well as institutional review boards at the Universities of Alabama at Birmingham, Northwestern University, and the Division of Research at Kaiser Permanente Health Care Plan. Written informed consent was obtained at every examination. Participants in this study were 90 controls selected from those who had fasting glucose <100 mg/dL at all 5 examinations (years 0, 7, 10, 15, and 20) at which glucose was measured. Controls were selected at random from each of several year 0 body mass index (BMI) categories (<20, 20–24.9, 25–29.9, 30–39.9, and 40+ kg/m2); these categories were chosen so that controls were frequency matched to cases.

### Measurement

Demographics, health behaviors, anthropometrics, and various clinical variables were measured at baseline and follow-up examinations. Blood samples were collected after an overnight fast. BMI was measured weight/height^2^ (kg/m^2^). Plasma total cholesterol, high-density lipoprotein cholesterol (HDL-C), and triglycerides were determined enzymatically by Northwest Lipids Research Laboratory (Seattle, Washington). Low-density lipoprotein cholesterol (LDL-C) was computed using the Friedewald equation. Fasting plasma glucose was determined using the hexokinase-ultraviolet method and fasting plasma insulin by radioimmunoassay (both at Linco Research Inc., St Louis, Missouri, USA). The homeostasis model assessment value for insulin resistance (HOMA–IR) was calculated as (glucose in mmol/L × insulin in µIU/mL)/22.5.

### POPs analyses

POPs were measured in stored 2 mL serum samples collected at year 2 (stored frozen at −70°C until analysis). The samples were analyzed by solid phase extraction and final determination using gas chromatography isotope dilution high-resolution mass spectrometry (GC/ID-HRMS) at the Centers for Disease Control and Prevention[13]. Laboratory personnel were masked to all CARDIA data, including case-control status. A total of 55 POPs were measured: 9 organochlorine (OC) pesticides, 35 polychlorinated biphenyl (PCB) congeners, 10 polybrominated diphenyl ether (PBDE) congeners, and 1 polybrominated biphenyl (PBB) congener (**Table S1**).

### Statistical methods

Lipid standardized concentrations, the concentration of serum POP value divided by total serum lipid content (mg/dL), have generally been reported in epidemiological studies, since POPs are predominantly carried in the lipid component of the blood. However, according to a simulation study[14], wet weight concentrations adjusting for triglycerides and total cholesterol had less bias than did lipid standardized concentrations. Thus, we presented wet weight concentrations adjusting for triglycerides and total cholesterol by including these two lipid profiles into the models as covariates. On the other hand, as POPs themselves can disturb lipid metabolism[9], [15], adjustment for serum lipid concentrations may represent over-adjustment. Even though not adjusting for triglycerides and total cholesterol led to stronger associations, we presented adjusted results as a conservative analysis. Furthermore adjustment for baseline values is needed so that the year 20 outcome value represents the evolution of the study outcomes over 18 years. Analyses were done across quartiles of individual POPs, with sample sizes in each quartile of each individual POP provided in **Table S2**. Only measurements with more than 75% of values above the detection limit (31 POPs: 8 OC pesticides, 22 PCBs, and 1 PBB congeners) were included, placing participants with non-detectable concentrations in the lowest quartile of each POP.

We used general linear models to explore the associations of serum concentrations of POPs at year 2 with BMI, triglycerides, HDL-cholesterol, LDL-cholesterol, and HOMA-IR at the year 20 examination. The covariates included age, sex, race, triglycerides and total cholesterol at year 2. Year 20 HDL-cholesterol and HOMA-IR were additionally adjusted for their baseline values at year 2 and year 7, respectively. We considered P<0.05 as statistically significant, but, considering the small sample size, we also comment on results with P_trend_ or P_quadratic_<0.10.

## Results

At baseline (year 2 of CARDIA, **Table 1**) the average age of these study subjects free of diabetes was 27.2±3.3 years (mean ± standard deviation). Men and whites were 31.1% and 51.1% of the sample, respectively. BMI was 29.1±6.7 kg/m^2^ at year 2; and 33.0±7.9 kg/m^2^ at year 20.

**Table 1:** Baseline characteristics of study subjects.

Characteristics	
Age (years)	27.2±3.3
Men, %	31.1
White, %	51.1
BMI (kg/m^2^)	29.1±6.7
Triglycerides (mg/dL)	78.4±46.4
HDL-cholesterol (mg/dL)	53.2±13.7
LDL-cholesterol (mg/dL)	111.5±32.9
Fasting glucose* (mg/dL)	87.5±6.5
Fasting insulin* (µIU/mL)	12.9±6.9
HOMA-IR* (mmol/L * µIU/mL)	3.1±1.7

*Baseline was CARDIA year 2, except for fasting glucose, fasting insulin, and HOMA-IR, which were measured at year 7.

Among various OC pesticides, the most statistically significant year 2 predictor of BMI at year 20, was p,p'-DDE (**Table 2**). However, an increase of >6 kg/m^2^ in mean BMI was observed only up to the 3^rd^ quartile of p,p'-DDE, while mean BMI decreased in the 4^th^ quartile (P_quadratic_<0.01). p,p'-DDT also appeared to predict BMI at year 20, but the dose-response curve was close to a linear increase (P_trend_ = 0.05). The associations between PCBs and BMI differed depending on PCB congener. For example, PCB170, PCB180, or PCB206 showed the highest increase of BMI in the 2^nd^ quartile, while BMI decreased at higher concentrations of these PCBs, making inverted U-shaped associations. However, PCB156, PCB194, and PCB209 showed decreasing trends of BMI from the 2^nd^ to 4^th^ quartile with little or no increase of BMI from the 1^st^ to 2^nd^ quartile.

**Table 2 pone-0015977-t002:** Adjusted* means of body mass index (kg/m^2^) at year 20 according to quartiles of organochlorine (OC) pesticides, polychlorinated biphenyl (PCBs) or polybrominated biphenyl (PBB).

	Quartiles of OC pesticides, PCBs, or PBB					
Compounds	Q1	Q2	Q3	Q4	P_trend_	P_quadratic_
***OC pesticides***						
Oxychlordane	32.0	35.5	32.2	32.4	0.68	0.17
* Trans*-nonachlor	31.8	34.5	32.7	33.0	0.79	0.37
Hexachlorobenzene	32.3	32.8	32.0	35.1	0.37	0.33
β-hexachlorocyclohexane	31.4	33.4	33.9	33.2	0.43	0.35
γ- hexachlorocyclohexane	32.5	32.7	32.3	34.6	0.34	0.42
p,p'-DDE	29.7	33.1	36.3	32.8	0.05	<0.01
p,p'-DDT	32.3	30.5	34.0	35.4	0.05	0.21
Mirex	34.0	31.0	34.4	31.6	0.59	0.89
***PCBs (number of*** ** chlorines)**						
PCB74 (4)	32.7	32.5	33.8	33.0	0.77	0.79
PCB87 (5)	33.9	32.2	33.5	32.4	0.66	0.82
PCB99 (5)	31.3	33.0	34.0	33.7	0.22	0.46
PCB105 (5)	31.9	34.0	32.6	33.6	0.63	0.74
PCB118 (5)	32.2	33.2	33.7	32.9	0.74	0.51
PCB146 (6)	33.9	34.3	33.0	30.7	0.13	0.33
PCB153 (6)	31.4	35.7	32.9	32.0	0.57	0.07
PCB156 (6)	34.3	35.1	33.0	29.7	0.02	0.13
PCB157 (6)	33.1	35.8	31.7	31.7	0.15	0.38
PCB138–158 (6)	31.4	35.3	31.3	34.0	0.82	0.71
PCB167 (6)	31.8	34.6	32.4	33.2	0.80	0.45
PCB170 (7)	32.5	35.3	33.5	30.6	0.19	0.04
PCB178 (7)	32.7	36.4	32.0	30.8	0.12	0.08
PCB180 (7)	32.8	36.7	32.1	30.3	0.06	0.03
PCB183 (7)	32.2	34.6	33.1	32.1	0.83	0.20
PCB187 (7)	32.6	36.8	31.6	30.9	0.10	0.09
PCB194 (8)	34.6	35.9	32.2	29.2	0.01	0.11
PCB195 (8)	32.5	35.5	33.0	31.0	0.22	0.08
PCB199 (8)	33.5	35.2	33.1	30.0	0.08	0.06
PCB196–203 (8)	33.4	36.1	32.6	29.8	0.05	0.05
PCB206 (9)	33.6	35.0	34.2	29.6	0.09	0.02
PCB209 (10)	34.3	34.6	33.0	30.1	0.05	0.24
***PBB***						
PBB153	34.6	32.4	34.1	30.9	0.15	0.69

*Adjusted for age, sex, race, BMI, triglyceride and total cholesterol at year 2.

Plasma triglycerides were 77.9±46.4 mg/dL at year 2 and 97.3±61.5 mg/dL at year 20. Among OC pesticides, oxychlordane, trans-nonachlor, hexachlorobenzene, and p'p-DDE predicted future triglycerides at year 20 after adjusting for triglycerides at year 2 (**Table 3**). Oxychlordane, trans-nonachlor, and p'p-DDE showed increase in triglycerides in the 2^nd^ quartile with lower triglycerides in either quartile 3 or 4, while hexachlorobenzene predicted triglycerides with a positive linear trend. Many PCBs among those with 7 or more chlorides showed statistically significant low dose effects, having inverted U shaped associations. Opposite to findings concerning highly chlorinated PCBs, PCB74 (with 4 chlorides) showed a U-shaped association.

**Table 3 pone-0015977-t003:** Adjusted* means of triglycerides (mg/dL) at year 20 according to quartiles of organochlorine (OC) pesticides, polychlorinated biphenyl (PCB) or polybrominated biphenyl (PBB).

	Quartiles of OC pesticides, PCBs, or PBB					
Compounds	Q1	Q2	Q3	Q4	P_trend_	P_quadratic_
***OC pesticides***						
Oxychlordane	68.3	94.6	89.5	89.3	0.17	0.04
*Trans*-nonachlor	71.4	93.1	91.8	84.6	0.28	0.04
Hexachlorobenzene	72.5	85.9	80.4	104.1	0.05	0.55
β-hexachlorocyclohexane	80.9	90.3	85.8	82.7	0.97	0.40
γ- hexachlorocyclohexane	78.8	95.4	80.8	85.4	0.95	0.46
p,p'-DDE	65.5	92.5	100.5	84.4	0.03	<0.01
p,p'-DDT	77.4	83.9	87.3	91.7	0.19	0.86
Mirex	85.8	90.4	83.0	82.0	0.65	0.76
***PCBs (number of chlorine)***						
PCB74 (4)	88.6	73.9	82.9	96.5	0.33	0.06
PCB87 (5)	82.7	76.0	97.5	83.9	0.42	0.70
PCB99 (5)	75.4	86.4	88.4	90.0	0.21	0.48
PCB105 (5)	80.8	85.8	91.3	82.1	0.75	0.33
PCB118 (5)	76.9	95.1	80.1	88.5	0.66	0.49
PCB146 (6)	78.1	86.9	91.9	82.8	0.70	0.23
PCB153 (6)	77.3	84.1	92.5	86.2	0.48	0.38
PCB156 (6)	84.0	89.4	82.8	83.7	0.73	0.78
PCB157 (6)	76.7	93.4	78.7	93.8	0.53	0.96
PCB138–158 (6)	73.3	88.5	85.2	93.8	0.15	0.60
PCB167 (6)	74.3	89.5	85.4	91.3	0.22	0.49
PCB170 (7)	77.5	87.6	88.2	86.6	0.63	0.42
PCB178 (7)	76.7	92.3	91.3	79.8	0.96	0.06
PCB180 (7)	78.2	89.8	85.8	86.0	0.76	0.43
PCB183 (7)	70.8	90.1	95.6	84.6	0.17	0.03
PCB187 (7)	74.8	91.9	94.3	79.5	0.90	0.03
PCB194 (8)	75.1	99.1	79.9	87.0	0.90	0.31
PCB195 (8)	77.2	95.0	87.6	80.7	0.88	0.11
PCB199 (8)	76.8	93.4	93.2	77.1	0.89	0.02
PCB196–203 (8)	75.5	95.1	86.2	83.5	0.87	0.14
PCB206 (9)	77.6	92.1	91.3	79.1	0.95	0.06
PCB209 (10)	86.0	97.6	84.3	73.5	0.18	0.12
***PBB***						
PBB153	84.8	80.0	100.1	76.3	0.69	0.22

*Adjusted for age, sex, race, BMI, triglyceride and total cholesterol at year 2.

Plasma HDL-cholesterol was 53.2±13.7 mg/dL at year 2 and 54.1±14.5 mg/dL at year 20. The only OC pesticide which significantly predicted HDL-cholesterol at year 20 was p,p'-DDE (**Table 4**). The decrease of HDL-cholesterol was already evident in the 2^nd^ quartile, with no further decrease in the 3^rd^ and 4^th^ quartiles of p,p'-DDE. All PCBs that had statistically significant associations with HDL-cholesterol showed low dose effects. However, unlike the findings with p,p'-DDE, after the initial decrease of HDL-cholesterol from the 1^st^ to 2^nd^ quartile, HDL-cholesterol increased from the 2^nd^ to 4^th^ quartile of these PCBs.

**Table 4 pone-0015977-t004:** Adjusted* means of HDL-cholesterol (mg/dL) at year 20 according to quartiles of organochlorine (OC) pesticides, polychlorinated biphenyl (PCB) or polybrominated biphenyl (PBB).

	Quartiles of OC pesticides, PCBs, or PBB					
Compounds	Q1	Q2	Q3	Q4	P_trend_	P_quadratic_
***OC pesticides***						
Oxychlordane	52.6	51.8	54.6	50.7	0.85	0.57
*Trans*-nonachlor	55.2	49.9	52.9	51.9	0.55	0.32
Hexachlorobenzene	52.6	51.6	54.3	51.2	0.98	0.64
β-hexachlorocyclohexane	55.7	48.3	54.8	51.4	0.72	0.37
γ- hexachlorocyclohexane	53.6	52.8	54.1	49.4	0.27	0.34
p,p'-DDE	61.5	49.9	49.7	49.6	<0.01	0.01
p,p'-DDT	53.1	52.7	54.5	49.6	0.41	0.32
Mirex	52.0	57.0	49.3	53.2	0.72	0.93
***PCBs (number of chlorines)***						
PCB74 (4)	50.2	55.5	51.8	52.3	0.91	0.33
PCB87 (5)	53.9	53.9	50.1	52.1	0.36	0.66
PCB99 (5)	53.1	55.7	50.8	50.1	0.19	0.47
PCB105 (5)	51.5	51.7	54.6	51.9	0.65	0.52
PCB118 (5)	49.7	55.1	52.3	52.8	0.65	0.26
PCB146 (6)	51.5	48.9	54.1	55.4	0.16	0.41
PCB153 (6)	56.0	47.7	52.8	53.8	0.68	0.05
PCB156 (6)	52.0	49.1	51.5	57.3	0.09	0.06
PCB157 (6)	53.9	48.3	51.5	56.1	0.26	0.03
PCB138–158 (6)	53.8	50.0	54.4	51.8	0.94	0.79
PCB167 (6)	52.7	50.9	53.9	52.3	0.87	0.96
PCB170 (7)	53.4	49.4	49.4	58.2	0.15	<0.01
PCB178 (7)	53.9	46.5	53.4	57.0	0.11	0.02
PCB180 (7)	52.7	47.9	51.3	58.7	0.05	<0.01
PCB183 (7)	52.1	51.2	52.8	53.8	0.61	0.67
PCB187 (7)	55.0	45.0	55.1	55.9	0.14	0.02
PCB194 (8)	50.5	48.1	51.9	60.1	0.01	0.02
PCB195 (8)	54.0	46.9	51.7	58.1	0.09	<0.01
PCB199 (8)	52.3	47.3	50.8	61.0	0.02	<0.01
PCB196–203 (8)	52.3	48.5	52.1	57.6	0.11	0.04
PCB206 (9)	53.6	47.5	49.1	59.7	0.12	<0.01
PCB209 (10)	53.9	48.7	52.2	55.1	0.54	0.08
***PBB***						
PBB153	55.3	49.7	52.2	52.8	0.74	0.15

*Adjusted for age, sex, race, BMI, triglyceride, total cholesterol, and HDL-cholesterol at year 2.

HOMA-IR increased over 20 years from 2.6±1.4 to 3.4±1.9 mmol/L * µIU/mL. p,p'-DDE also predicted HOMA-IR at year 20 (**Table 5**). Insulin resistance increased by the 3^rd^ quartile of p,p-DDE and did not further increase at higher p,p;-DDE concentrations. PCBs with 5 chlorides such as PCB87 and PCB99 linearly predicted HOMA-IR while PCBs with 7 or 8 chlorides showed inverted U-shaped associations.

**Table 5:** Adjusted* means of HOMA-IR (µIU/mL * mmol/L/22.5) at year 20 according to quartiles of organochlorine (OC) pesticides, polychlorinated biphenyl (PCB) or polybrominated biphenyl (PBB).

	Quartiles of OC pesticides, PCBs, or PBB					
Compounds	Q1	Q2	Q3	Q4	P_trend_	P_quadratic_
***OC pesticides***						
Oxychlordane	3.0	3.6	2.6	3.0	0.55	0.65
Trans-nonachlor	2.6	3.9	2.7	3.2	0.72	0.34
Hexachlorobenzene	3.4	2.7	2.9	3.3	0.85	0.12
β-hexachlorocyclohexane	2.7	3.2	2.9	3.5	0.23	0.96
γ- hexachlorocyclohexane	2.7	3.4	2.9	3.3	0.46	0.66
p,p'-DDE	2.3	3.1	3.6	3.3	0.02	0.06
p,p'-DDT	2.9	2.7	3.2	3.4	0.25	0.67
Mirex	3.0	3.3	3.1	2.9	0.85	0.51
***PCBs (number of chlorine)***						
PCB74 (4)	2.9	2.6	3.3	3.5	0.17	0.55
PCB87 (5)	2.8	2.6	3.3	3.6	0.08	0.42
PCB99 (5)	2.5	2.7	3.4	3.7	0.01	0.90
PCB105 (5)	2.8	2.8	3.1	3.5	0.19	0.68
PCB118 (5)	2.8	2.8	2.8	3.8	0.08	0.19
PCB146 (6)	3.2	3.0	3.0	3.1	0.91	0.71
PCB153 (6)	2.6	3.5	2.8	3.2	0.69	0.52
PCB156 (6)	3.2	3.4	2.8	2.8	0.25	0.78
PCB157 (6)	3.0	3.6	2.5	3.2	0.61	0.66
PCB138–158 (6)	2.7	3.0	2.7	3.8	0.13	0.22
PCB167 (6)	2.7	3.2	2.7	3.7	0.19	0.48
PCB170 (7)	2.7	3.5	3.1	2.8	0.63	0.13
PCB178 (7)	2.8	3.8	3.0	2.6	0.34	0.04
PCB180 (7)	2.8	3.7	2.9	2.8	0.41	0.13
PCB183 (7)	2.8	3.1	3.0	3.3	0.39	0.98
PCB187 (7)	2.8	3.8	2.9	2.8	0.54	0.17
PCB194 (8)	3.0	4.1	2.6	2.5	0.07	0.16
PCB195 (8)	2.9	3.8	3.0	2.6	0.27	0.11
PCB199 (8)	3.1	3.7	3.1	2.4	0.12	0.06
PCB196–203 (8)	2.9	3.9	3.0	2.5	0.16	0.03
PCB206 (9)	3.4	3.7	2.7	2.6	0.10	0.62
PCB209 (10)	3.4	3.4	2.7	2.7	0.18	0.99
***PBB***						
PBB153	3.4	3.1	3.1	2.6	0.17	0.72

*Adjusted for age, sex, race, BMI, triglyceride, and total cholesterol at year 2, and HOMA-IR at year 7.

Calculated LDL-cholesterol was associated with only 3 POPs (hexachlorobenzene, PBB153, and PCB199) with slightly different dose-response curves (**Table S3**).

**Figure 1:**
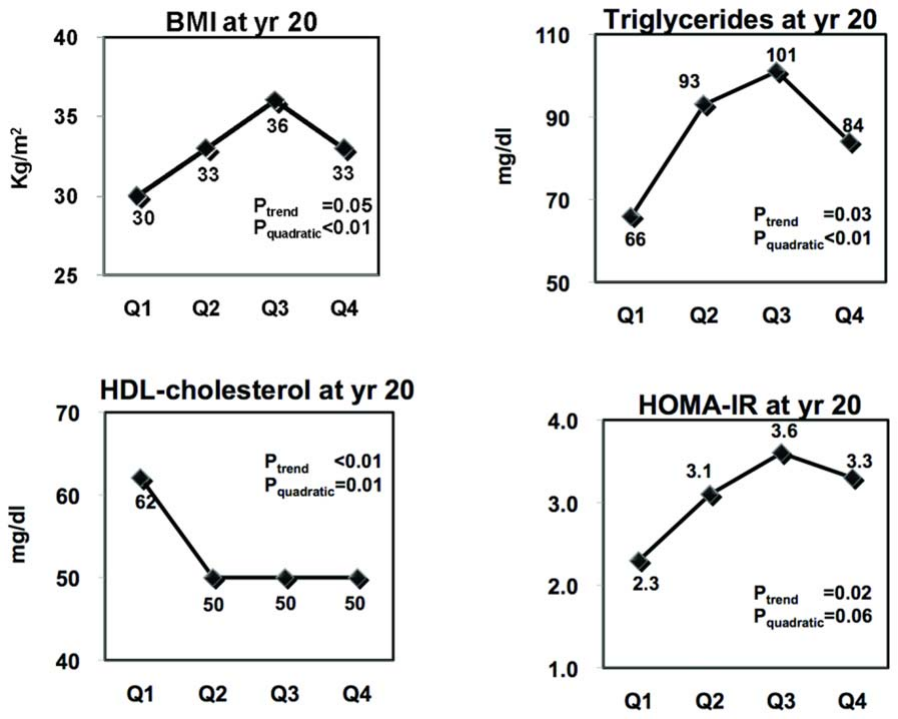
Effects of p,p'-DDE on BMI, dyslipidemia, and insulin resistance. Adjusted means of year 20 BMI, triglycerides, HDL-cholesterol, and HOMA-IR according to serum concentrations of p,p'-DDE at year 2. Adjusting variables were age, sex, race, BMI, triglycerides, and total cholesterol at year 2. Year 20 HDL-cholesterol and HOMA-IR were additionally adjusted for their baseline values at year 2 and year 7, respectively.

**Figure 2:**
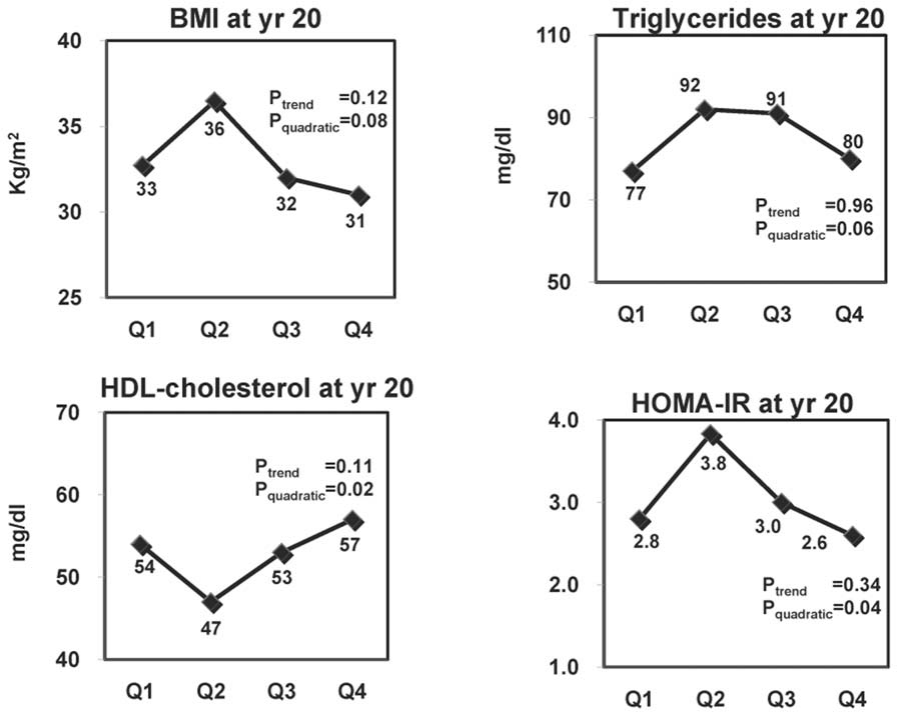
Effects of PCB178 on BMI, dyslipidemia, and insulin resistance. Adjusted means of year 20 BMI, triglycerides, HDL-cholesterol, and HOMA-IR according to serum concentrations of PCB178 at year 2. Adjusting variables were age, sex, race, BMI, triglycerides, and total cholesterol at year 2. Year 20 HDL-cholesterol and HOMA-IR were additionally adjusted for their baseline values at year 2 and year 7, respectively.

For illustrative purposes, we displayed the results on p, p'-DDE and PCB178 in **Figure 1** **and** **Figure 2**; these POPs predicted most metabolic profiles and pictorialize some of the kinds of dose-response curves reported in the tables. We also displayed the consistency across several POPs associations that were significant or marginally significant (p_trend_ or p_quadratic_<0.1) in **Tables 1** **,** **2** **,** **3** **,** **4** and in **Figure S1 and Figure S2**.

## Discussion

In this prospective study, some OC pesticides and PCBs predicted excess adiposity, dyslipidemia, and insulin resistance among participant*s* without diabetes. Similar to observed risk for type 2 diabetes[1], low dose OC pesticides and PCBs showed strong associations, often with inverted U-shaped associations.

The past two decades have observed an explosive worldwide increase in the number of type 2 diabetic patients,with obesity regarded as a primary cause. Metabolic changes precede the development of type 2 diabetes by years or even decades. Insulin resistance is regarded as the initial metabolic defect, and dyslipidemia is also commonly observed. At first, the pancreatic beta-cells are able to compensate by increasing insulin secretion, but, as insulin resistance worsens, type 2 diabetes occurs when the pancreas fails to sustain this compensatory activity. Our study showed that POPs are likely involved in all these steps of pathogenesis of type 2 diabetes, even including the development of obesity. Importantly, different kinds of POPs were differentially associated with various aspects of metabolic dysregulation. For example, p,p'-DDE predicted increased BMI, increased triglycerides, decreased HDL-cholesterol, and increased HOMA-IR in this study, while p,p'-DDE did not predict type 2 diabetes in the previous study[1]. On the other hand, trans-nonachlor was most strongly associated with the development of diabetes, but not BMI, HDL-cholesterol, and HOMA-IR although trans-nonachlor predicted triglycerides. Therefore, p,p'-DDE may be influential in development of prediabetic conditions, while trans-nonachlor may be more important in the progression from insulin resistance to type 2 diabetes.

An important finding in this study was that some POPs predicted future BMI. Although imbalance between energy intake and expenditure is the central mechanism leading to obesity, the recent surge of obesity might have other causes besides overeating and physical inactivity. In addition to POPs[11], several selected environmental pollutants such as diethylstilbesterol, bisphenol A, phthalate, or tributyltin that cause perturbations in endogenous hormonal regulation involved in weight homeostasis have been recently studied as possible risk factors for obesity[16], [17], [18] although most studies have been performed in animals, focusing on the fetal exposure to chemicals. Chemicals may cause obesity by altering homeostatic metabolic set-points, disrupting appetite controls, perturbing lipid homeostasis to promote adipocyte hypertrophy, or stimulating adipogenic pathways that enhance adipocyte hyperplasia during development or in adults[16], [17]. This study showed that low dose exposure of adults to p,p'-DDE, p,p'-DDT, and some PCBs with 7 or more chlorines predicted future BMI. In fact, earlier animal experiments consistently observed that many chemicals cause weight loss at high exposure, but much lower concentrations of these same chemicals are weight-promoting[19].

Among the 31 POPs we studied, 10 POPs statistically significantly or marginally significantly predicted future higher triglycerides and 14 POPs predicted lower HDL-cholesterol. However, higher LDL-cholesterol was associated with only 3 compounds. Lipid disorders are common in type 2 diabetes and play a crucial role in the development of diabetic cardiovascular complications. In fact, high triglycerides and low HDL-cholesterol, often with normal LDL-cholesterol, are fundamental characteristics of the metabolic syndrome [20].

Despite the general occurrence in these findings of inverted U-shaped associations, some dose-response curves followed a different pattern, depending on outcome and/or specific POP. Among OC pesticides, hexachlorobenzene predicted future triglycerides and LDL-cholesterol linearly, while p,p-DDE, oxychlordane, and trans-nonachlor showed low dose effects. Dose-response curves also differed between moderately chlorinated and highly chlorinated PCBs. In the case of moderately chlorinated PCBs such as PCB87, PCB99, and PCB118, the evolution of HOMA-IR occurred above the 3^rd^ quartile. However, in the case of highly chlorinated PCBs, such as PCB178, PCB194, and PCB199, mean HOMA-IR increased from the 1^st^ to 2^nd^ quartile and then clearly decreased through the 4^th^ quartile, forming inverted U-shaped associations.

Similar to the associations between low dose POPs and type 2 diabetes, underlying mechanisms for associations between POPs and disturbances in glucose and lipid metabolism have been little studied. As PCB congeners with affinity to Aryl hydrocarbon receptor (AhR), like PCB105, PCB118, and PCB167, were not associated with any outcome in this study, affinity to AhR may not play an important mechanistic role in the metabolic disturbances studied here. Also, estrogenic activity is unlikely to be critical because both estrogenic PCB187 and antiestrogenic PCB170 showed similar associations. In addition, although p,p-DDT has a weak-estrogenic activity, p,p'-DDE, the OC pesticide which was strongly associated with most outcomes in this study and the most persistent metabolite of commercial DDT, is an anti-androgen rather being a weak estrogen[21]. The only common property of PCB groups which predicted BMI, dyslipidemia, or HOMA-IR appeared to be their long persistency because the persistence of PCB congeners increases with the number of chlorines[22].

The findings on POPs observed in both current and previous studies may help to explain the current world-wide epidemic of metabolic syndrome and type 2 diabetes. The disturbances of glucose and lipid metabolism due to POPs seem to require at least two conditions; low dose and persistency. As serum concentrations of most chlorinated POPs have decreased during several decades, low doses of POPs among our study subjects which were measured in stored serums in 1987 to 1988 were similar to the highest concentrations currently seen in the general population. A very long exposure to POPs may be another critical condition. Because POPs have been released into the environment since the 1950s, we can assume that our study subjects had lifetime exposure to these chemicals. Therefore, in terms of both exposure dose and duration, the current general population may be at higher risk of developing glucose and lipid dysmetabolism due to POPs than were earlier general populations. In addition, obesity appeared to worsen the problem, because the associations between individual POPs and type 2 diabetes were observed only among obese persons in our previous study. Also, the exposure to xenobiotics such as POPs may contribute to the development of obesity. Finally, findings observed in man may be explained by the effects of individual POPs working together. Combinations of endocrine disrupting chemicals are able to produce significant effects, even when each chemical is present at low doses that individually do not induce observable effects[23]. As the general population is simultaneously exposed to a mixture of several hundred POPs, a cocktail effect due to mixed POPs may be important.

A limitation of this study is potentially false positive associations among the many associations evaluated. We assessed the possible effect of multiple testing under a binomial model by counting the number of significant findings (p<0.05) among the 62 linear or quadratic tests performed for each outcome, compared to 3.1 tests (95% confidence interval 0 to 6.5) expected to achieve p<0.05 under the null hypothesis. As 11 tests with BMI, 7 tests with triglyceride, and 15 tests with HDL-cholesterol achieved statistical significance, many of the associations of POPs with BMI, triglyceride, and HDL-cholesterol observed in the current study were unlikely under the null hypothesis, even considering multiple testing. As only 3 of 62 tests concerning LDL-cholesterol achieved statistical significance, multiple testing might well explain these apparently significant tests. The situation concerning evaluation of HOMA-IR is more complex. Although only 4 of 62 tests achieved statistical significance, the range of HOMA-IR was constrained by excluding anyone with impaired fasting glucose over 20 years. This exclusion restricts glucose explicitly and insulin secondarily. Furthermore, the significant associations that were observed were with p,p'-DDE and PCB178, both consistently showed significant associations with BMI, triglycerides, and HDL-cholesterol. Metabolic syndrome links BMI, triglycerides, and HDL-cholesterol with HOMA-IR, so that it is physiologically reasonable to expect similar associations of POPs with each of these variables. Therefore, the findings concerning p,p'-DDE and PCB178 with HOMA-IR should not simply be dismissed due to multiple testing. Furthermore, great consistency in prediction was observed for highly chlorinated PCBs. Another study limitation was small sample size, which was a compromise related to practicalities of cost and use of precious samples. Volumes of the available blood samples also prevented us from measuring dioxins and furans. The primary strength of the study is the long term followup, with baseline measures of all outcome variables.

In conclusion, low dose POPs may be involved in the development of obesity, dyslipidemia, and insulin resistance, although different POPs may relate to different metabolic traits. As the general population is simultaneously exposed to various POPs through food consumption, our findings concerning POPs could help to explain why these metabolic abnormalities tend to occur as a cluster. Together with the findings on type 2 diabetes in our previous report[1], the background exposure to POPs may help to explain the recent epidemic of obesity, metabolic syndrome, and diabetes.

## Supporting Information

Figure S1 Means of year 20 BMI, triglyceride, HDL-cholesterol, and HOMA-IR according to quartiles of selected organochlorine pesticides which showed Ptrend<0.1 or Pquadratic<0.1 in Tables 1, 2, 3, 4 and 5. Adjusted for age, sex, race, BMI, triglycerides, and total cholesterol at year 2. Year 20 HDL-cholesterol and HOMA-IR were additionally adjusted for their baseline values at year 2 and year 7, respectively.
(PDF)

Figure S2 Means of year 20 BMI, triglyceride, HDL-cholesterol, and HOMA-IR according to quartiles of selected PCBs which showed Ptrend<0.1 or Pquadratic<0.1 in the Tables 1 to [Table pone-0015977-t002] [Table pone-0015977-t003] [Table pone-0015977-t004] 5. Adjusted for age, sex, race, BMI, triglycerides, and total cholesterol at year 2. Year 20 HDL-cholesterol and HOMA-IR were additionally adjusted for their baseline values at year 2 and year 7, respectively.
(PDF)

Table S1 Target analytes with detection rate and abbreviations used.
(DOCX)

Table S2 Number of subjects in each quartile of organochlorine (OC) pesticides, polychlorinated biphenyl (PCB) or polybrominated biphenyl (PBB).
(DOCX)

Table S3 Adjusted* means of LDL-cholesterol (mg/dL) at year 20 according to quartiles of organochlorine (OC) pesticides, polychlorinated biphenyl (PCB) or polybrominated biphenyl (PBB).
(DOCX)
