# Supplementary material for: Low Dose Organochlorine Pesticides and Polychlorinated Biphenyls Predict Obesity, Dyslipidemia, and Insulin Resistance among People Free of Diabetes
Source: PLoS One. 2011 Jan 26;6(1):e15977. doi: 10.1371/journal.pone.0015977 (PMC3027626; doi:10.1371/journal.pone.0015977)

Figure S1. Means of year 20 BMI, triglyceride, HDL-cholesterol, and HOMA-IR according to quartiles of selected organochlorine pesticides which showed $P_{trend}<0.1$ or $P_{quadratic}<0.1$ in Tables 1 to 5. Adjusted for age, sex, race, BMI, triglycerides, and total cholesterol at year 2. Year 20 HDL-cholesterol and HOMA-IR were additionally adjusted for their baseline values at year 2 and year 7, respectively. (Note that triglycerides means are nearly coincident for hexachlorobenzene and p.p'-DDE.)

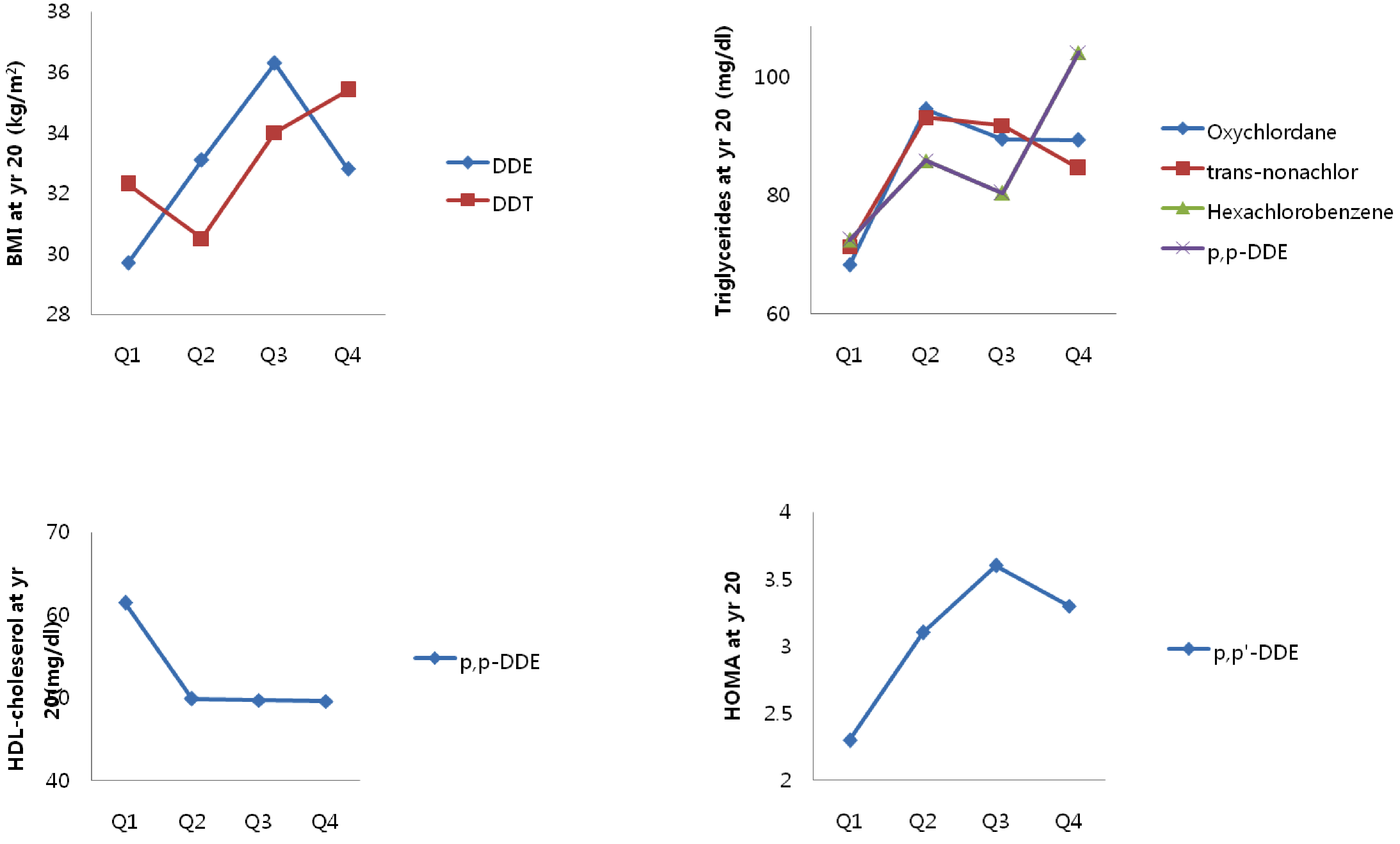

Supplement: Figure S1 — Means of year 20 BMI, triglyceride, HDL-cholesterol, and HOMA-IR according to quartiles of selected organochlorine pesticides which showed Ptrend<0.1 or Pquadratic<0.1 in Tables 1, 2, 3, 4 and 5. Adjusted for age, sex, race, BMI, triglycerides, and total cholesterol at year 2. Year 20 HDL-cholesterol and HOMA-IR were additionally adjusted for their baseline values at year 2 and year 7, respectively. (PDF) [file pone.0015977.s001.pdf]
