# Supplementary material for: Low Dose Organochlorine Pesticides and Polychlorinated Biphenyls Predict Obesity, Dyslipidemia, and Insulin Resistance among People Free of Diabetes
Source: PLoS One. 2011 Jan 26;6(1):e15977. doi: 10.1371/journal.pone.0015977 (PMC3027626; doi:10.1371/journal.pone.0015977)
Supplement: Table S1 — Target analytes with detection rate and abbreviations used. (DOCX) [file pone.0015977.s003.docx]

Supplementary table 1. Target analytes with detection rate and abbreviations used

| Target analytes * | Detection rate | Abbreviations |
| --- | --- | --- |
| Organochlorine (OC) Pesticides |  |  |
| Hexachlorobenzene | 100% |  |
| β-Hexachlorocyclohexane | 98% |  |
| γ-Hexachlorocyclohexane (Lindane) | 87% |  |
| Oxychlordane | 99% |  |
| Trans-Nonachlor | 100% |  |
| 2,2-Bis(4-chlorophenyl)-1,1-dichloroethene | 100% | p,p’-DDE |
| 2-(4-chlorophenyl)-2-(2-chlorophenyl)- 1,1,1-trichloroethan | 44% | o,p’-DDT |
| 2,2-Bis(4-chlorophenyl-1,1,1-trichloroethan | 100% | p,p’-DDT |
| Mirex | 75% |  |
| Polychlorinated Biphenyl (PCB) congener |  |  |
| 2,4,4’-trichlorobiphenyl | 9% | PCB28 |
| 2,2’,3,5’-tetrachlorobiphenyl | 14% | PCB44 |
| 2,2’,4,5’-tetrachlorobiphenyl | 7% | PCB49 |
| 2,2’,5,5’-tetrachlorobiphenyl | 33% | PCB52 |
| 2,3’,4,4’-tetrachlorobiphenyl | 63% | PCB66 |
| 2,4,4’,5-tetrachlorobiphenyl | 100% | PCB74 |
| 2,2’,3,4,5’-pentachlorobiphenyl | 78% | PCB87 |
| 2,2’,4,4’,5-pentachlorobiphenyl | 100% | PCB99 |
| 2,2’,4,5,5’-pentachlorobiphenyl | 58% | PCB101 |
| 2,3,3’,4,4’-pentachlorobiphenyl | 100% | PCB105 |
| 2,3,3’,4’,6-pentachlorobiphenyl | 49% | PCB110 |
| 2,3’,4,4’,5-pentachlorobiphenyl | 100% | PCB118 |
| 2,2’,3,3’,4,4’-hexachlorobiphenyl | 50% | PCB128 |
| 2,2’,3,4,4’,5’-hexachlorobiphenyl and 2,3,3’,4,4’,6-hexachlorobiphenyl | 100% | PCB138-158 |
| 2,2’,3,4’,5,5’-hexachlorobiphenyl | 96% | PCB146 |
| 2,2’,3,4’,5’,6-hexachlorobiphenyl | 60% | PCB149 |
| 2,2’,3,5,5’,6-hexachlorobiphenyl | 58% | PCB151 |
| 2,2’,4,4’,5,5’-hexachlorobiphenyl | 100% | PCB153 |
| 2,3,3’,4,4’,5-hexachlorobiphenyl | 100% | PCB156 |
| 2,3,3’,4,4’,5’-hexachlorobiphenyl | 98% | PCB157 |
| 2,3’,4,4’,5,5’-hexachlorobiphenyl | 95% | PCB167 |
| 2,2’,3,3’,4,4’,5-heptachlorobiphenyl | 100% | PCB170 |
| 2,2’,3,3’,4,5,5’-heptachlorobiphenyl | 64% | PCB172 |
| 2,2’,3,3’,4’,5,6-heptachlorobiphenyl | 97% | PCB177 |
| 2,2’,3,3’,5,5’,6-heptachlorobiphenyl | 94% | PCB178 |
| 2,2’,3,4,4’,5,5’-heptachlorobiphenyl | 100% | PCB180 |
| 2,2’,3,4,4’,5’,6-heptachlorobiphenyl | 100% | PCB183 |
| 2,2’,3,4’,5,5’,6-heptachlorobiphenyl | 100% | PCB187 |
| 2,3,3’,4,4’,5,5’-heptachlorobiphenyl | 48% | PCB189 |
| 2,2’,3,3’,4,4’,5,5’-octachlorobiphenyl | 99% | PCB194 |
| 2,2’,3,3’,4,4’,5,6-octachlorobiphenyl | 94% | PCB195 |
| 2,2’,3,3’,4,4’,5’,6-octachlorobiphenyl and 2,2’,3,4,4’,5,5’,6-octachlorobiphenyl | 100% | PCB196-203 |
| 2,2’,3,3’,4,5,6,6’-octachlorobiphenyl | 100% | PCB199 |
| 2,2’,3,3’,4,4’,5,5’,6-nonachlorobiphenyl | 100% | PCB206 |
| 2,2',3,3',4,4',5,5',6,6'decachlorobiphenyl | 97% | PCB209 |
| Polybrominated Diphenyl Ether (PBDE) congeners |  |  |
| 2,2',4- tribromodiphenyl ether | 3% | PBDE-17 |
| 2,4,4’-tribromodiphenyl ether | 5% | PBDE-28 |
| 2,2’,4,4’-tetrabromodiphenyl ether | 11% | PBDE-47 |
| 2,3',4',4-tetrabromodiphenyl ether | 2% | PBDE-66 |
| 2,2’,3,4,4’-pentabromodiphenyl ether | 8% | PBDE-85 |
| 2,2’,4,4’,5-pentabromodiphenyl ether | 9% | PBDE-99 |
| 2,2’,4,4’,6-pentabromodiphenyl ether | 19% | PBDE-100 |
| 2,2’,4,4’,5,5’-hexabromodiphenyl ether | 100% | PBDE-153 |
| 2,2’,4,4’,5,6’-hexabromodiphenyl ether | 6% | PBDE-154 |
| 2,2’,3,4,4’,5’,6-heptabromodiphenyl ether | 12% | PBDE-183 |
| Polybrominated Biphenyl (PBB) congener |  |  |
| 2,2’,4,4’,5,5’-hexabromobiphenyl | 100% | PBB-153 |

* : POPs with detection rate ≥ 75% were included in final analyses
