# Supplementary material for: Low Dose Organochlorine Pesticides and Polychlorinated Biphenyls Predict Obesity, Dyslipidemia, and Insulin Resistance among People Free of Diabetes
Source: PLoS One. 2011 Jan 26;6(1):e15977. doi: 10.1371/journal.pone.0015977 (PMC3027626; doi:10.1371/journal.pone.0015977)
Supplement: Table S2 — Number of subjects in each quartile of organochlorine (OC) pesticides, polychlorinated biphenyl (PCB) or polybrominated biphenyl (PBB). (DOCX) [file pone.0015977.s004.docx]

Supplementary table 2. Number of subjects in each quartile of organochlorine (OC) pesticides, polychlorinated biphenyl (PCB) or polybrominated biphenyl (PBB)

|  | Quartiles of OC pesticides, PCBs, or PBB | | | |
| --- | --- | --- | --- | --- |
| Compounds | Q1 | Q2 | Q3 | Q4 |
| ***OC pesticides*** |  |  |  |  |
| Oxychlordane | 22 | 23 | 23 | 22 |
| *Trans*-nonachlor | 22 | 23 | 23 | 22 |
| Hexachlorobenzene | 22 | 23 | 23 | 22 |
| β-hexachlorocyclohexane | 22 | 23 | 23 | 22 |
| γ- hexachlorocyclohexane | 22 | 23 | 23 | 22 |
| p,p’-DDE | 22 | 23 | 23 | 22 |
| p,p’-DDT | 22 | 23 | 23 | 22 |
| Mirex | 29 | 16 | 23 | 22 |
| ***PCBs (number of chlorine)*** |  |  |  |  |
| PCB74 (4) | 22 | 23 | 23 | 22 |
| PCB87 (5) | 22 | 22 | 24 | 22 |
| PCB99 (5) | 22 | 23 | 23 | 22 |
| PCB105 (5) | 23 | 22 | 23 | 22 |
| PCB118 (5) | 22 | 23 | 23 | 22 |
| PCB146 (6) | 22 | 22 | 24 | 22 |
| PCB153 (6) | 22 | 23 | 23 | 22 |
| PCB156 (6) | 23 | 22 | 22 | 23 |
| PCB157 (6) | 23 | 21 | 24 | 22 |
| PCB138-158 (6) | 22 | 23 | 23 | 22 |
| PCB167 (6) | 22 | 23 | 23 | 22 |
| PCB170 (7) | 22 | 23 | 23 | 22 |
| PCB178 (7) | 22 | 23 | 23 | 22 |
| PCB180 (7) | 22 | 23 | 23 | 22 |
| PCB183 (7) | 22 | 23 | 23 | 22 |
| PCB187 (7) | 22 | 23 | 23 | 22 |
| PCB194 (8) | 22 | 23 | 23 | 22 |
| PCB195 (8) | 22 | 22 | 24 | 22 |
| PCB199 (8) | 22 | 23 | 23 | 22 |
| PCB196-203 (8) | 22 | 23 | 23 | 22 |
| PCB206 (9) | 21 | 23 | 22 | 23 |
| PCB209 (10) | 23 | 22 | 23 | 22 |
| ***PBB*** |  |  |  |  |
| PBB153 | 22 | 23 | 23 | 22 |
