# Supplementary material for: Low Dose Organochlorine Pesticides and Polychlorinated Biphenyls Predict Obesity, Dyslipidemia, and Insulin Resistance among People Free of Diabetes
Source: PLoS One. 2011 Jan 26;6(1):e15977. doi: 10.1371/journal.pone.0015977 (PMC3027626; doi:10.1371/journal.pone.0015977)
Supplement: Table S3 — Adjusted* means of LDL-cholesterol (mg/dL) at year 20 according to quartiles of organochlorine (OC) pesticides, polychlorinated biphenyl (PCB) or polybrominated biphenyl (PBB). (DOCX) [file pone.0015977.s005.docx]

Supplementary table 3. Adjusted* means of LDL-cholesterol (mg/dL) at year 20 according to quartiles of organochlorine (OC) pesticides, polychlorinated biphenyl (PCB) or polybrominated biphenyl (PBB)

|  | Quartiles of OC pesticides, PCBs, or PBB | | | |  |  |
| --- | --- | --- | --- | --- | --- | --- |
| Compounds | Q1 | Q2 | Q3 | Q4 | P_trend_^†^ | P_quadratic_^†^ |
| ***OC pesticides*** |  |  |  |  |  |  |
| Oxychlordane | 106.2 | 93.1 | 107.7 | 103.1 | 0.76 | 0.33 |
| *Trans*-nonachlor | 92.7 | 103.5 | 109.8 | 103.7 | 0.20 | 0.15 |
| Hexachlorobenzene | 98.4 | 93.3 | 103.1 | 117.5 | 0.04 | 0.10 |
| β-hexachlorocyclohexane | 103.9 | 101.8 | 99.2 | 104.8 | 0.97 | 0.53 |
| γ- hexachlorocyclohexane | 111.0 | 98.1 | 98.4 | 102.8 | 0.42 | 0.14 |
| p,p’-DDE | 100.2 | 96.9 | 115.1 | 97.3 | 0.74 | 0.23 |
| p,p’-DDT | 108.0 | 96.3 | 101.4 | 104.0 | 0.88 | 0.20 |
| Mirex | 98.5 | 96.8 | 107.9 | 105.8 | 0.28 | 0.90 |
| ***PCBs (number of chlorine)*** |  |  |  |  |  |  |
| PCB74 (4) | 97.8 | 102.2 | 101.2 | 108.9 | 0.32 | 0.80 |
| PCB87 (5) | 95.8 | 108.1 | 100.8 | 105.4 | 0.43 | 0.52 |
| PCB99 (5) | 99.5 | 105.1 | 101.4 | 103.3 | 0.83 | 0.74 |
| PCB105 (5) | 102.9 | 98.9 | 99.9 | 108.5 | 0.59 | 0.29 |
| PCB118 (5) | 103.0 | 102.1 | 99.8 | 104.7 | 0.94 | 0.64 |
| PCB146 (6) | 107.1 | 98.4 | 107.7 | 96.0 | 0.47 | 0.77 |
| PCB153 (6) | 102.7 | 93.5 | 114.5 | 99.3 | 0.68 | 0.64 |
| PCB156 (6) | 107.1 | 99.6 | 102.2 | 100.3 | 0.76 | 0.65 |
| PCB157 (6) | 107.2 | 100.6 | 98.7 | 102.8 | 0.73 | 0.37 |
| PCB138-158 (6) | 100.7 | 102.6 | 103.6 | 102.4 | 0.85 | 0.79 |
| PCB167 (6) | 94.9 | 104.7 | 105.6 | 104.4 | 0.36 | 0.33 |
| PCB170 (7) | 100.3 | 97.1 | 113.9 | 98.8 | 0.70 | 0.33 |
| PCB178 (7) | 100.5 | 99.0 | 111.7 | 97.9 | 0.87 | 0.27 |
| PCB180 (7) | 102.6 | 101.6 | 107.1 | 97.9 | 0.75 | 0.49 |
| PCB183 (7) | 94.9 | 104.5 | 106.3 | 103.7 | 0.34 | 0.28 |
| PCB187 (7) | 102.1 | 99.6 | 110.8 | 96.5 | 0.83 | 0.34 |
| PCB194 (8) | 103.1 | 100.9 | 106.6 | 98.6 | 0.83 | 0.61 |
| PCB195 (8) | 99.0 | 101.4 | 108.5 | 99.7 | 0.77 | 0.30 |
| PCB199 (8) | 91.5 | 106.8 | 113.2 | 98.0 | 0.50 | <0.01 |
| PCB196-203 (8) | 99.9 | 100.0 | 109.6 | 99.5 | 0.89 | 0.37 |
| PCB206 (9) | 94.7 | 103.1 | 108.4 | 103.5 | 0.34 | 0.25 |
| PCB209 (10) | 100.1 | 96.5 | 106.8 | 106.5 | 0.42 | 0.78 |
| ***PBB*** |  |  |  |  |  |  |
| PBB153 | 93.6 | 98.4 | 110.4 | 108.2 | 0.07 | 0.56 |

* : Adjusted for age, sex, race, BMI, triglyceride, total cholesterol, and LDL-cholesterol at year 2
